# Making Sense of Modeling in Elementary Literacy Instruction

**DOI:** 10.1002/trtr.1863

**Published:** 2019-08-12

**Authors:** Kristine M. Schutz, Emily C. Rainey

**Keywords:** Strategies, methods, and materials, Instructional strategies, teaching strategies < Strategies, methods, and materials, Teacher education, professional development, Preservice < Teacher education, professional development, Instructional strategies; methods and materials, Teacher education; professional development, 5‐College/university students, 6‐Adult, 2‐Childhood

## Abstract

Although modeling is an instructional approach commonly named in literacy education circles, the authors struggled to articulate the essential features of modeling to preservice teachers. This was a problem for them and for the preservice teachers with whom they worked. The problem also represents a larger one in the field, which is that educators are still building that which is the foundation of most other professions: a shared professional language. Efforts to build a shared professional language are important for literacy educators seeking to reflect on and improve their craft, literacy leaders working to make change at the school level, and mentor teachers and teacher educators tasked with preparing the next generation of teachers. The authors describe their efforts to articulate and represent modeling in elementary literacy instruction.

Delve into important components of modeling for literacy teaching in elementary classrooms through multiple representations of the instructional practice for preservice teachers.

**Pause and Ponder**

■ When you think of modeling, what do you think of?
■ How do you model key strategies and practices in your professional context?
■ How did you learn to model? How do you teach others to model?

Our field is awash with terms that people use to mean different things. Consider current buzzwords such as *project‐based teaching*,* inquiry learning*, and *cultural relevance*. Although most literacy educators are very familiar with these terms, as a field, we cannot yet expect that everyone who uses these terms has the same conception of what they do and do not involve. This lack of a shared professional language poses challenges for educators who are striving to improve their craft, groups of educators who are working to make change at the school level, and mentor teachers and teacher educators who are striving to support the preparation of the next generation of teachers (Ball & Forzani, 2009).

When we began working together in 2011 as teacher educators, we had this very problem. We both had backgrounds as literacy teachers and teacher educators; we were steeped in scholarship about the importance of scaffolding young students’ reading, composing, and reasoning; and we were interested in helping new teachers learn to model as part of their elementary literacy instruction. However, we frequently found ourselves using different language in our attempt to describe what modeling for literacy learning involved. Was modeling the same as thinking aloud? Could it involve simply demonstrating something? Did it matter whether the teacher or students were sharing the work? Did it matter if there was a physical artifact produced? With some humility, we acknowledged that any attempts to help novice teachers learn to see and enact the fundamental aspects of modeling while simultaneously honoring its complexity would be limited by our own abilities to name them for ourselves.

In this article, we describe our journey to come to a common understanding of how we might represent modeling to elementary preservice teachers. Although we never expected that we would generate a single best way of modeling, we conducted our work assuming that there were likely some common features of the instructional approach that could be studied and named. Classroom teachers may find our way of parsing the instructional practice of modeling helpful as they aim to reflect on and improve their own teaching practice or that of their grade‐level team. Our representations of modeling for preservice teachers are likely to be useful for university‐based teacher educators, along with the many readers of *The Reading Teacher* who are also teacher educators or coaches, such as those serving as mentors of teacher candidates or district/school instructional coaches. Also, more broadly, we hope that by sharing our process of studying and reconciling multiple meanings of modeling, we can offer ideas to others seeking to deeply understand or refine instructional practice and the professional language we use to describe it.

## What Is Modeling? Why Does It Matter?

There is a strong literature base in support of modeling as an effective approach to literacy instruction in classrooms. For more than 40 years, literacy researchers and educators have been amassing knowledge about the value of explicit literacy instruction for young learners (Au & Raphael, 1998; Duffy, 2009; Duffy & Roehler, 1987; Englert, Raphael, Anderson, Anthony, & Stevens, 1991; Graham, Harris, & Chambers, 2016; Palincsar & Brown, 1984; Paris, Lipson, & Wixson, 1983; Pearson & Gallagher, 1983). Modeling—the instructional practice in which teachers represent the invisible work involved in reading, writing, and reasoning with texts—has been studied and advanced as a means for providing explicit literacy instruction (e.g., Dunn, 2011; Fisher, Frey, & Lapp, 2008, 2011; Taylor, Peterson, Pearson, & Rodriguez, 2002).

Duke and Pearson (2002) described modeling as providing a broken‐apart model of the process or work of making meaning from text. For them, modeling is both demonstrating and thinking aloud to make a process visible so students can learn to engage in the same process. The approach of modeling for students is often situated within a gradual release of responsibility framework (Pearson & Gallagher, 1983), in which teacher‐driven efforts support students’ effort until students reach independence. Flexible and responsive applications of the gradual release of responsibility situate the use of teacher modeling in response to learners’ needs (Webb, Massey, Goggans, & Flajole, 2019). Modeling does not always begin a lesson. Rather, it is used as teachers recognize the specific needs of learners as they engage in activity. The implicit goal is that overt strategies will eventually go underground and transform from strategies to skills as readers engage with them fluidly, without even noticing (Afflerbach, Pearson, & Paris, 2008).

However, as clear as the research is about the potential power of modeling for literacy learning, this body of work does not fully help teachers know what to do when they are modeling. How are teachers, especially novice teachers, to know when they are getting it right? How are instructional coaches to know exactly what to look for when conducting an observation or suggesting next steps to a colleague? How are teacher educators to know whether their preservice teachers are on their way to learning to model effectively?

## We Cannot Teach What We Cannot Name

We brought a commitment to practice‐based teacher education to this work. Practice‐based teacher education seeks to develop ways of supporting adults to learn professional practice through mediated cycles of studying, trying out, and reflecting on bounded activities or routines (McDonald, Kazemi, & Kavanagh, 2013). It is based in understandings that teaching is contextually sensitive and embodied work and that learning to teach involves supported practical activity toward particular goals. Rather than casting the work of teaching as technical, discrete, acontextual sets of moves to be learned and implemented, most scholars working in practice‐based teacher education seek to develop new ways of supporting preservice teachers toward highly responsive, relational, sophisticated practice.

Grossman et al. (2009) conducted an empirical study of how professions other than teaching support their novices into professional practice. They focused on two professions that, like teaching, are dynamic, relational, and require sophisticated judgment: clinical psychology and the clergy. They found that those professions had systemic ways of preparing adults for those professional roles: professional educators working in clinical psychology programs and seminaries decomposed highly complex work by chunking it into parts with names and descriptions; they represented highly complex work by showing videos, live examples, and written transcripts so the complex activity could be slowed down and studied; and they approximated practice to provide scaffolded opportunities for learners to try out what they were learning and receive feedback before they worked with actual clients or parishioners.

Since 2009, teacher educators and education researchers have worked to borrow from and adapt these approaches for teacher professional learning. So, we are joined by many colleagues who have sought to specify and parse instructional routines and moves to make the complexity of teacher practice more learnable (cf. Alston, Danielson, Dutro, & Cartun, 2018; Kucan & Palincsar, 2011).

Along with many of our colleagues, we are deeply aware of the risks of the enterprise to decompose, represent, and approximate teaching practice for teacher learning. A commonly articulated concern is that decomposition reifies and oversimplifies the inherently complex, situated work of teaching. However, there are also risks of not attending to the practice of teaching through developing shared language and approaches, including the risk that teaching is understood as idiosyncratic and potentially absent of features strongly linked to student learning.

## Process for Naming Components of Modeling

Building on ongoing efforts to attend more squarely to practice while supporting teachers, we sought to name important components of modeling. Our ultimate goal was to support preservice elementary teachers to learn to enact high‐quality modeling in their literacy instruction.

The work is drawn from a larger design‐based research effort focused on better understanding instructional practice across subject areas and grade levels (Ball & Forzani, 2010). As a part of that work, we studied 10 first‐year elementary teachers who volunteered to participate (Schutz, Rainey, & Iwashyna, 2014). We video recorded each teacher as they enacted a literacy lesson that included modeling with their students in their classroom. In this phase of data collection, each teacher was encouraged to design and teach as they typically would. Teachers then engaged in a modeling simulation. In this phase, each teacher was provided a scenario describing a fictionalized group of learners. The scenario asked participants to model a specific strategy for adding details to a personal narrative. We designed the fixed‐scenario task as a way of giving ourselves the best chance of seeing commonalities in the teachers’ moves, and we included observation of teachers’ actual teaching practice because we understood that teaching is contextually specific, responsive, and relational work.

We sought to describe modeling through analyzing these recorded classroom observations and simulations. An early step of this process involved making judgments about which episodes of modeling were the most likely to actually help students learn a literacy skill or strategy if they did not already use it with some level of independence. A way that we reduced the number of episodes to analyze was to ask ourselves, As an adult observing in this classroom, am I clear about what students are supposed to be doing and how they are supposed to be doing it? Are students supposed to be learning a literacy skill or strategy? Where we answered no to these questions, we removed those videos from our data set.

From there, we used constant comparative analysis (Glaser, 1965) to iteratively break apart the data and begin to detect patterns across the episodes of teaching. As we viewed each video, we began a running list of features that we noticed. This list included multiple grain sizes, including instructional moves such as highlighting a core idea, framing the work, and using precise language. Then, we sought to look across the various codes to organize them and detect patterns across them. Our understanding of the literature summarized in the previous sections, along with our own research and teaching experiences as both classroom teachers and teacher educators, informed our thinking as we analyzed videos of teaching and attempted to come to a common understanding of important elements of modeling.

## Naming Components of Modeling

Our analysis of teaching videos revealed three interrelated aspects of modeling for literacy learning: showing, situating, and abstracting. We discovered that teachers seemed to engage these aspects of modeling recursively. Rather than moving in a sequence from one step to the next, teachers moved fluidly among showing, situating, and abstracting.

### Showing

When we observed teachers showing as part of their literacy modeling, they made important aspects of a focal strategy or practice visible to students. When showing, teachers would actually show the doing work (e.g., reading excerpts of the text where a repeated image or object was mentioned, crossing out repetitive words while revising text, pointing and looking closely at an illustration that accompanied the print). Teachers would also show the thinking work that one engages in when using the strategy. For example,

I'm thinking to myself, Hmm, what could they mean? What could the author be wanting me to think of when I read about those tall boots? So, here the tall boots are coming into this beautiful scene…so that's telling me this is ominous. It makes me feel hopeless.

Essentially, teachers gave learners a glimpse into the inner monologue of their minds.

### Situating

When we observed teachers situating as part of their literacy modeling, they introduced and connected a focal strategy or practice to students’ recent learning or experiences. For example, a fourth‐grade teacher situated by connecting the focal strategy that students were to learn with an earlier discussion that had been held in class:

A few days ago, we talked about Dumbo, and I talked to you about how that little feather represented his courage and his strength and his self‐confidence. When he had the feather, he was able to fly. I find that movies have these really powerful images that symbolize big ideas. Today I want to use “The Butterfly” to talk about symbolism. You might have noticed that there are some really powerful images in this book. And today, I want to show you how I as a reader can find symbols.

Here, the teacher is situating because she connects students’ earlier experience learning about symbolism to this lesson. Situating also involved other sorts of framing moves that put the process to be learned into a familiar context for students, and it involved describing a purpose for using a focal strategy while reading or writing.

### Abstracting

When we observed teachers abstracting, they pulled out of the immediate task being modeled to name and distill what students were learning to do in similar but not identical tasks or contexts. Specific moves included using marking language to narrate their process (i.e., the thinking work, the doing work) and to highlight necessary components (e.g., “So, what you just saw me do there was…,” “Watch closely as I…”) and tucking in helpful pointers to help students in their early attempts using strategies or processes (e.g., “Remember, it's not cheating to look at those illustrations. They often carry meaning that the words don't”). Ultimately, abstracting appeared to be the aspect of modeling that made the strategies transferable as it disentangled the process from the immediate context of the modeling.

## Representing Modeling as a Rich Instructional Practice

Our analysis yielded observable components of modeling and some useful examples of moments of each component from various episodes of modeling. Because our participants were first‐year teachers, our analysis also indirectly affirmed that modeling is an instructional practice used by teachers and that at least some first‐year teachers are modeling in ways that are well described by this framework.

Yet, we puzzled over how to represent modeling—as a whole, but simultaneously making the components from our decomposition visible—to novices. We realized that if we were to help preservice teachers learn to design and enact the type of modeling we saw some of our teacher participants doing, then we would need to be careful about how we represented these three parts. Our field has a history of breaking down many literacy teaching routines into ordered steps. For instance, calling attention to the types of instruction that students need before, during, and after reading (Fountas & Pinnell, 2017) has been very important for influencing teaching practice. Similarly, individual instructional activities such as a writing conference can be thought of as a chronological sequence of activity in which the teacher researches, decides, compliments, teaches, and links (Calkins, Hartman, & White, 2005).

Although it may be helpful to parse some literacy teaching routines and practices into steps, we decided that it could be misleading to represent modeling that way based on the fluidity of modeling we observed. Thus, we explored various other ways of representing these three aspects to support adult learners.

### Representation 1

The first representation we created is a framework that articulates the components and key features of each component of modeling (see Table 1). By representing modeling as having three major component parts and then offering a shorthand for what is included in each component part, we sought to support preservice teachers to reflect on instances of modeling (while observing instruction or reflecting on their own past instruction) in a more specific and nuanced way. This tool was also meant to support novices to design new lessons that would support students’ development of literacy strategies and skills.

**Table 1:** Aspects of Modeling

Showing	Situating	Abstracting
■ Show the doing work: Apply the strategy to a prepared or preselected text. ■ Show the thinking work: Think aloud in a focused way about what is going on in your mind as you enact the strategy. ■ Produce an artifact (when applicable).	■ Name and explain the literacy strategy/practice: What is it called? What does it involve? ■ Connect the focal strategy/practice to students’ recent literacy learning, lives, or interests: How does it connect with prior instruction or experiences? ■ Describe a purpose for using this strategy/practice that relates to students’ recent literacy learning.	■ Label and explain key features of this instance of modeling to support students to apply the strategy/practice with flexibility themselves. ■ Use marking language to signal a shift from showing to abstracting (e.g., “Did you see how I first ___ and then ___?”; “Watch me as I ___”; “You should try to ___ and ___ in your reading later today”). ■ Note other contexts in which the focal strategy could be useful.

### Representation 2

**Figure 1:**
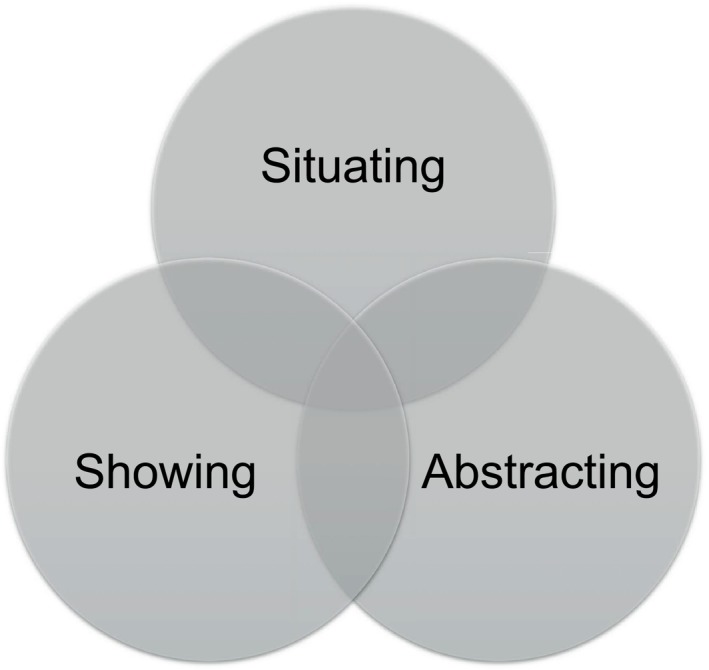
Modeling as Bringing Together Interrelated Parts

Because the framework in representation 1 might be misunderstood as a sort of checklist of discrete behaviors or actions rather than a description of a fluid instructional practice, we also developed variations of images to show the interrelation between the aspects (see Figure 1 for an example). Representations such as this Venn diagram make clear that there is not a linear order of steps to follow; instead, modeling involves helping students understand how what they are learning is connected to their prior learning, offering them a chance to see the invisible thinking work happening, and supporting them to notice key parts of the work that they should attend to in their own activity. It is important to note that even though these representations name the teacher moves of modeling, consideration of students and the learning context are required as a part of those moves. One cannot situate a focal lesson without reflecting on what students’ opportunities to learn have entailed and the cultural, linguistic, and literate resources they bring to their learning, and one cannot abstract without deeply considering the sorts of literate and linguistic practices that students are typically engaging in.

### Representation 3

**Figure 2:**
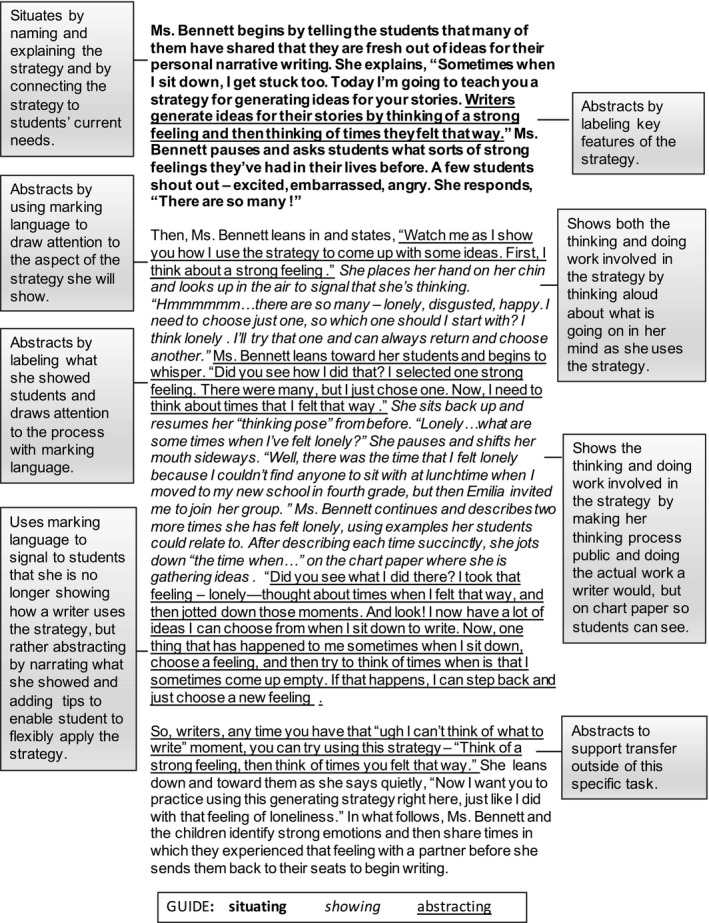
Annotated Vignette

We also puzzled over how to provide exemplars to preservice teachers that were still within their reach. The most sophisticated examples of modeling that we collected moved so quickly among the three components and did so with such nuance that we worried that novices would not be able to detect those moves in video or transcribed text. Other observations that we conducted did not include all three parts, or included them but in ways that were somewhat misaligned. In the spirit of rendering complex instructional practice learnable, we created a vignette inspired by the instruction we had studied. Figure 2 is an annotated vignette in which a fictional second‐grade teacher, Ms. Bennett, models how to generate ideas for writing by identifying a strong feeling and thinking of times you felt that way (Serravallo, 2017) at the start of a writing unit on personal narratives.

When shared in written form, the annotations enable teachers to begin to develop a common language to describe modeling and its component parts. In our work with preservice teachers, we have modified its use in two ways. First, we demonstrate the modeling as if we were Ms. Bennett and discuss the demonstration, drawing attention to the features of modeling in our framework. Then, we provide an unannotated vignette and ask teachers to mark up places where the teacher was situating, showing, and abstracting. Finally, preservice teachers have an opportunity to try on the vignette themselves with a partner. Of course, this work of slowing down and developing practical moves is not a full approximation of teaching, but it serves useful instructional purposes for adult learning.

### Representation 4

Old knowledge influences new learning. Prior conceptions about modeling can limit our abilities to attend to different ways of specifying the practice. To support the unlearning process, we created contrasting examples of modeling to make visible potentially absent or problematic aspects, drawing from the trends we have noted while working with novice teachers in the past. For example, sometimes we noticed that preservice teachers would say that they were modeling, but they were only explaining a strategy and describing an example of its use. Other times, preservice teachers would say that they were modeling when they shared two different drafts, a before and after, which they had prepared ahead of time instead of creating in front of learners. In Table 2, we share an example and two nonexamples of modeling that we have used to highlight how the form of modeling we are putting forth differs from the conceptions of modeling that preservice teachers may bring to their professional learning.

**Table 2:** Side‐by‐Side Representations of Modeling: Example and Nonexamples

Modeling example that involves showing, situating, and abstracting
Yesterday as I listened in as you shared your personal narratives with partners, I noticed something. You've been working really hard at helping your readers envision settings and characters in your stories, and it's making them more interesting. Today, I'm going to teach you one more way writers add detail to their stories, in particular about their characters. Writers add details about their characters by showing instead of telling what these characters are like or how they feel. Writers do this by thinking about how their own bodies feel or what they say or do when they have the same feeling as the character, and then they put that in their writing.Watch me as I try that. So, right here, I have a spot where I wrote “Ronny was mad.” This was the spot where everyone was being picked for a team, and he and Nelson still hadn't been chosen. Instead of just saying that he was mad, I can try to show this feeling. Watch as I think about how my body might feel as I'm angry. [closes eyes and takes a deep breath while pursing lips together tightly, as if she is going to explode, then stomps foot] Gosh, I might feel so fed up at that point and angry that I hadn't been chosen that I'd scream, “I've had enough!” and even throw down my hat.Did you see what I did there? I actually tried to feel what Ronny was feeling in that moment and consider what I'd say or do. Once I've got that figured out, then I need to jot down that thinking in my draft. So, look here. I'll cross off the sentence “Ronny was mad.” [crosses off sentence] Then [revising text live in front of students], I'd insert “Ronny stomped his feet and threw down his hat. He had had enough!” Let me go back and read that. [rereads the sentence leading up to the revision and the new parts]You can do this, too. Just find a spot where you told how a character was feeling: He was mad, she was tired, they were frustrated. Then, think about what a character who felt that way might say or do, and write that down.

Modeling nonexample 1	Modeling nonexample 2
*Pitfall:* Explaining the strategy and describing an example	*Pitfall:* Sharing before and after instead of doing the work
One thing you can do to make your writing come to life for readers is to “show, don't tell” how characters are feeling. So, instead of saying, “Ronny was mad,” you might say, “Ronny stomped his feet and threw down his hat. He had had enough!” You can do this, too. Just find a spot where you told how a character was feeling: He was mad, she was tired, they were frustrated. Then, think about what a character who felt that way might say or do, and write that down. Got it? Now, you try!	So, look here at my draft before I tried using the “show, don't tell” strategy. [points to chart paper with draft] You see that I basically just said how my character felt: “Ronny was mad.” Well, then I decided to try to “show, don't tell,” and look what I wrote instead: “Ronny stomped his feet and threw down his hat. He had had enough!” Do you see what I did there? I thought about what Ronny might do to show that he was mad that he hadn't yet been chosen for a team.

Side‐by‐side representations enable differences to become more visible. Neither nonexample includes showing the doing and thinking work involved in applying the strategy. The teacher in nonexample 1 explains to students how one might use a strategy; the teacher in nonexample 2 shows students a teacher‐created draft before and after applying a strategy. Both of these versions leave out the important in‐the‐moment work of showing students the actual process of revision.

## Affordances of Decomposing and Representing Instructional Practice

Just as young learners may benefit from opportunities to see, consider, and try out ways of engaging in more complex literate practice, especially when those conditions are designed to promote learning, so too do adult learners benefit from opportunities to see, consider, and try out complex teaching practice. Also, just as young learners may benefit from instruction that supports their concept development, their development and use of a shared language for abstract processes and practices, and the guidance of an expert other, so too may adult learners benefit from opportunities to learn professional concepts and use and co‐construct a shared professional language mediated by a colleague, coach, or teacher educator.

We offer the decomposition and representations in this article as an example of a potentially promising on‐ramp for supporting preservice teachers to model for literacy learning. Although we acknowledge that parsing modeling does in some ways simplify the work, we maintain that it also has the potential to make complex practice more learnable for novice teachers, especially when used as part of a designed professional preparation program that attends to adults’ learning over time and adjusts by expanding and complexifying both what is to be learned and what is to be seen in practice.

We underscore that there is not just one way to decompose practice, and in many ways, the specific parsing is less important than the shared learning that these sorts of tools enable adults to do together. Our parsing was grounded in an analysis of observed teaching practice and enabled us to begin to describe and represent modeling in more accessible ways. We encourage other teacher educators to consider how our process of observing and studying instances of a particular instructional routine, generating clear labels for important aspects of that routine, and then collecting and creating examples may be useful in other professional contexts. Such efforts are likely helpful for developing a clear professional language locally (or possibly more broadly), for conducting professional development and coaching with teachers and preservice teachers, and for engaging in ongoing learning with colleagues.

## Notes

The data collection and analysis reported herein was funded by the Bill & Melinda Gates Foundation (grant OPP1053743). Any opinions, findings, conclusions, or recommendations expressed in this material are those of the authors and do not necessarily reflect the views of the funders. The ideas in this article originated from a larger research project involving faculty, graduate students, and others at TeachingWorks (http://www.teachingworks.org).

**TAKE ACTION!**

In addition to seeking to improve their own practice, many teachers are also teacher educators in some way. Here are some ideas that you can use to study and grow your own instructional approaches to modeling or support others to do so:

1. *Watch and notice:* View and deconstruct instances of modeling either live or via video.
2. *Write it out:* Script portions of modeling, paying particular attention to the concise and economical use of language.
3. *Practice:* Rehearse modeling, focusing on aspects that feel unfamiliar or uncomfortable. Get feedback from colleagues before working with students.
4. *Coplan and coteach:* Collaboratively design and teach a lesson that involves modeling for literacy learning. Invite others to watch and discuss this teaching.
